# Isolation of dimorphic chloroplasts from the single-cell C_4 _species *Bienertia sinuspersici*

**DOI:** 10.1186/1746-4811-8-8

**Published:** 2012-03-06

**Authors:** Shiu-Cheung Lung, Makoto Yanagisawa, Simon DX Chuong

**Affiliations:** 1 Department of Biology, University of Waterloo, 200 University Avenue West, Waterloo, Ontario N2L 3G1, Canada

**Keywords:** *Bienertia sinuspersici*, Chloroplast isolation, Dimorphic chloroplasts, Osmotic swelling, Photosynthesis, protoplast, Single-cell C_4_, Vacuole isolation

## Abstract

Three terrestrial plants are known to perform C_4 _photosynthesis without the dual-cell system by partitioning two distinct types of chloroplasts in separate cytoplasmic compartments. We report herein a protocol for isolating the dimorphic chloroplasts from *Bienertia sinuspersici*. Hypo-osmotically lysed protoplasts under our defined conditions released intact compartments containing the central chloroplasts and intact vacuoles with adhering peripheral chloroplasts. Following Percoll step gradient purification both chloroplast preparations demonstrated high homogeneities as evaluated from the relative abundance of respective protein markers. This protocol will open novel research directions toward understanding the mechanism of single-cell C_4 _photosynthesis.

## Background

The majority of terrestrial plants house chloroplasts primarily in one major cell type of leaves (i.e. mesophyll cells), and perform C_3 _photosynthesis to assimilate atmospheric CO_2 _into a 3-carbon product, 3-phosphoglyceric acid. In C_4 _species, on the other hand, a Kranz-type leaf anatomy featuring the second type of chlorenchyma cells surrounding the vascular bundles (i.e. bundle sheath cells) was reported as early as in the late 1800's [1]. In these species, the initial carbon fixation into 4-carbon acids was first documented in the 1960's [2,3]. The physiological relevance of the Kranz anatomy in relation to the C_4 _photosynthetic pathways, however, had not been elucidated until the successful separation of the two types of chlorenchyma cells and their respective dimorphic chloroplasts. With the development of various mechanical and enzymatic methods for separating the mesophyll and bundle sheath cells, the biochemistry of C_4 _cycles has been intensively studied over the past few decades focusing explicitly on characterizing the enzymatic properties and determining their precise subcellular locations in these cell types (for review, see [4]), leading to the current C_4 _models. In the C_4 _model, atmospheric CO_2 _is initially converted into C_4 _acids by phosphoenolpyruvate carboxylase (PEPC) in mesophyll cells. The C_4 _acids are broken down by a C_4 _subtype-specific decarboxylation enzyme in bundle sheath cells, and the liberated CO_2 _is subsequently re-fixed by ribulose-1,5-bisphosphate carboxylase/oxygenase (Rubisco). The C_4 _pathway concentrates CO_2 _at the site of Rubisco and minimizes the photorespiration process, an unfavorable oxygenase activity of Rubisco with O_2_.

The indispensable relationship between the Kranz anatomy and C_4 _photosynthesis has been an accepted feature until the discovery of three terrestrial single-cell C_4 _species, *Suaeda aralocaspica *(formerly called *Borszczowia aralocaspica*) [5], *Bienertia cycloptera *[6,7], and *B. sinuspersici *[8] in the Chenopodiaceae family. In chlorenchyma cells of these succulent Chenopodiaceae species, the C_4 _cycles are operational in the absence of Kranz anatomy due to the division of cytoplasm into two compartments. The cytoplasmic channels that connect the two compartments not only allow metabolite exchange but also limit inter-compartmental gas diffusion, resembling the function of plasmodesmata traversing the thickened and sometimes suberized bundle sheath cell wall. The two cytoplasmic compartments house two distinct chloroplast types and different subsets of enzymes, respectively. Accordingly, the peripheral compartment proximal to the CO_2 _entry point is specialized for carboxylation and regeneration of the initial carbon acceptor, phosphoenolpyruvate, whereas the central compartment distal to the CO_2 _entry point is responsible for decarboxylation of C_4 _acids and Rubisco-catalyzed re-fixation of the liberated CO_2_. In agreement with the immunolocalization patterns of the major enzymes involved [5,7,9], the two cytoplasmic compartments appear to be functionally equivalent to the mesophyll and bundle sheath cells of Kranz-type C_4 _plants, respectively. Based on differential speed centrifugation for the enrichment of each chloroplast type in subcellular fractions of *B. sinuspersici *leaves, Offermann et al. [10] have recently examined the protein distribution patterns of dimorphic chloroplasts and confirmed their functional similarities to the mesophyll and bundle sheath cells of Kranz-type C_4 _plants, respectively. Thorough studies of the enzymology of the single-cell C_4 _model, however, requires homogenous preparations of the dimorphic chloroplasts with specific techniques based on the unique cell anatomy, in analogy with the previous efforts of separation and subcellular fractionation of the dual cell types from Kranz-type C_4 _plants (for review, see [4]).

Here, we present our empirically optimized protocol for separating the dimorphic chloroplasts from chlorenchyma protoplasts of *B. sinuspersici*. By reducing the osmotic potential of culture medium to a suitable level, the isolated protoplasts were hypo-osmotically bursted, concomitantly extruding one type of chloroplasts encased in the central cytoplasmic compartment and another type of chloroplasts from the peripheral compartment adhered to the external surface of intact vacuoles. Following a subsequent centrifugation step, these two structures can be separated into the sedimented and floating fractions, respectively. Finally, two homogenous populations of dimorphic chloroplasts with minimal cross-contamination can be further purified by using a Percoll gradient. Overall, this dimorphic chloroplast isolation protocol can be applied in multiple areas of research toward further understanding the development of single-cell C_4 _systems.

## Results

### Rationale of the isolation procedures in relation to cell anatomy

**Figure 1:**
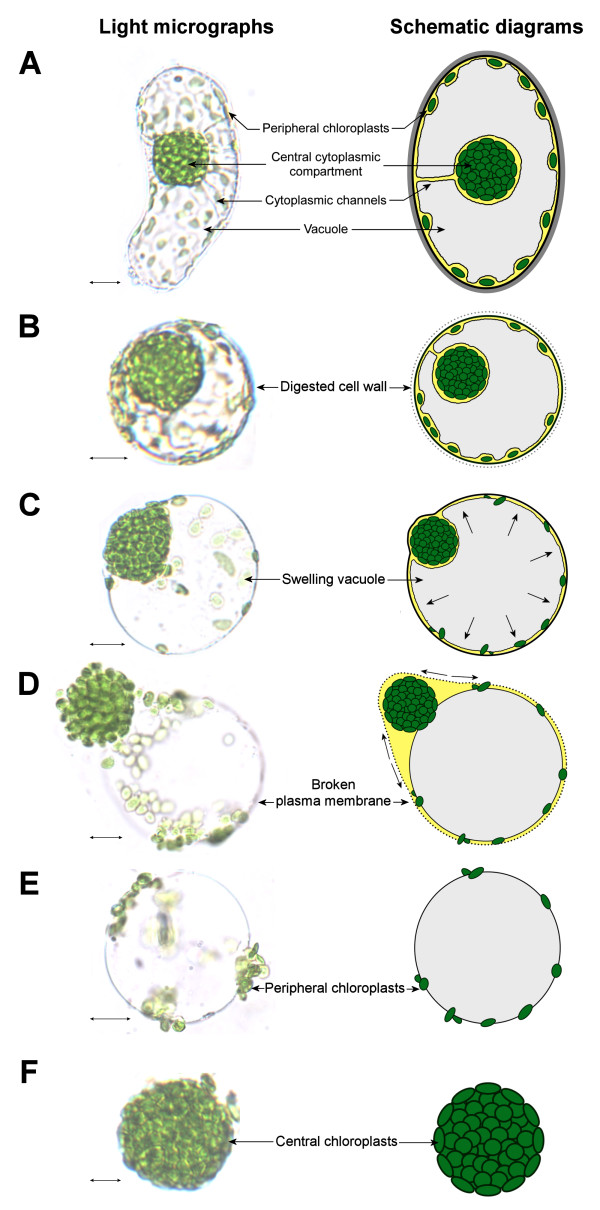
**Illustration of the cellular and subcellular morphologies during the process of dimorphic chloroplast isolation**. (**A**) A mature chlorenchyma cell. For clarity, only one cytoplasmic channel is illustrated despite the multiple occurrences in an actual cell. (**B**) An isolated protoplast with digested cell wall. (**C**) A swelling protoplast with increased osmotic pressure of the cell sap as indicated by arrows. (**D**) A broken protoplast with torn plasma membrane as indicated by arrows. (**E**) An intact vacuole carrying peripheral chloroplasts on the external surface. (**F**) An isolated central cytoplasmic compartment. *Scale bars *= 10 μm (A-E) or 5 μm (F).

For a better clarification of the each isolation step as described in the subsequent sections, we first summarize the major changes in the complex subcellular organization of chlorenchyma cells throughout the process as illustrated with schematic diagrams (Figure 1). In a mature chlorenchyma cell, a large central vacuole (as depicted in grey) separates the cytoplasm (yellow) into the peripheral (PCC) and the central (CCC) cytoplasmic compartments, which are interconnected by cytoplasmic channels traversing the vacuole (Figure 1A). The rounding of protoplasts by enzymatic digestion of the cell wall causes no changes in the integrity of the two cytoplasmic compartments and the cytoplasmic channels (Figure 1B). Reducing the osmotic potential of the cell-stabilizing buffer induces the swelling of the protoplast and its central vacuole, disrupting the cytoplasmic channels and pushing the CCC against the plasma membrane (Figure 1C). The hypo-osmotic shock causes the plasma membrane to stretch beyond its extension limit that eventually breaks, releasing the intact CCC and vacuole with attached peripheral chloroplasts (P-Chls; Figure 1D). This is in agreement with the previous observation showing that the two cytoplasmic compartments are separated by a single vacuole [9,11]. Eventually, the central vacuole carrying P-Chls on the external surface (Figure 1E) and the isolated CCC containing central chloroplasts (C-Chls; Figure 1F) can be separated into two discrete entities.

### Isolation of chlorenchyma protoplasts from *B. Sinuspersici*

**Figure 2:**
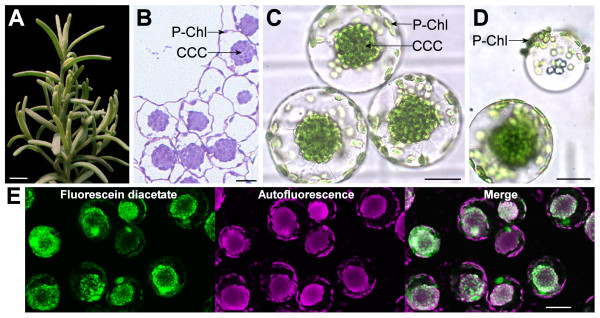
**Isolation of chlorenchyma protoplasts from *B. sinuspersici***. (**A**) A healthy branch of vegetatively propagated *B. sinuspersici *suitable for protoplast isolation. (**B**) A cross section of *B. sinuspersici *leaf showing chlorenchyma cells with two types of chloroplasts, i.e. the peripheral chloroplasts (P-Chls) in the peripheral cytoplasmic compartments and the central chloroplasts enclosed in the central cytoplasmic compartments (CCC). (**C**) A bright field image of chlorenchyma protoplasts indicating the integrity of the two cytoplasmic compartments for subsequent isolation of the dimorphic chloroplasts. (**D**) A bright field image of a protoplast and a vesicle with externally associated P-Chls. (**E**) Vital staining of isolated protoplasts imaged under epifluorescence microscopy. Fluorescein diacetate staining (*left panel*), chlorophyll autofluorescence (*middle panel*) and a merged image (*right panel*). *Scale bars *= 10 mm (**A**) or 20 μm (B-E).

As a first step, we isolated chlorenchyma cells from *B. sinuspersici *leaves and enzymatically prepared a homogenous population of healthy protoplasts. Interested readers should refer to our previous report for detailed technical considerations on plant growth conditions and chlorenchyma protoplast isolation [12]. Previously, progressive developmental variation has been identified at different stages of *B. sinuspersici *leaves in terms of the unique subcellular compartmentation and photosynthetic gene expression [11,13]. Similar developmental gradients were also observed across the base-to-tip dimension of leaves [13]. Thus, these major sources of variability were inevitably taken into considerations in order to standardize the degree of C_4 _functionality of the starting materials for dimorphic chloroplast isolation. To this end, we routinely propagated *B. sinuspersici *by vegetative cuttings and collected entire leaves, 2 cm or longer in length, from healthy branches of 3- to 4-month-old plants (Figure 2A). At this stage, the single-cell C_4 _compartmentation has reached maturity as evident by the presence of the distinctive subcellular distribution of dimorphic chloroplasts in the majority of chlorenchyma cells (Figure 2B). In a mature chlorenchyma cell, the C-Chls are densely packed into a large, spherical CCC structure, whereas the P-Chls are distributed throughout the thin layer of cytoplasm, (PCC; Figure 2B). Following cell wall removal by cellulase treatment under our optimized conditions, the resulting protoplasts exhibited no observable changes in their unique chloroplast distribution, which is considered a prerequisite for subsequent preparations of pure dimorphic chloroplasts (Figure 2C). During the protoplast isolation, we occasionally observed vesicles with externally adhering P-Chls (Figure 2D). Given their potential of serving as an excellent source of pure P-Chls, we continued to optimize the conditions for inducing the formation of these P-Chl-containing vesicles from isolated protoplasts. To guarantee intactness and full functionality of the purified dimorphic chloroplasts, at least 90% viability of the isolated protoplasts should be achieved prior to chloroplast separation as evaluated by fluorescein diacetate staining (Figure 2E).

### Hypo-osmotic shock treatment of isolated protoplasts induced vesicle formation

**Figure 3:**
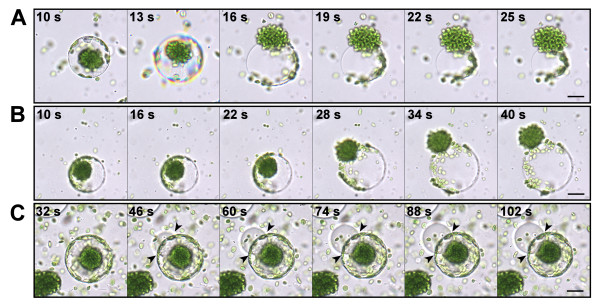
**Time-lapse imaging of protoplasts subjected to osmotic swelling**. (**A**) Diluting the cell stabilizing buffer to a sucrose concentration of 0.1 M led to rapid swelling and bursting of both the protoplasts and their vacuoles. (**B**) Lowering the osmotic potential of the cell stabilizing buffer to the optimum 0.2 M sucrose resulted in protoplast lysis and the release of intact central cytoplasmic compartments and vacuoles with attached peripheral chloroplasts. (**C**) Subtle dilution of the cell stabilizing buffer to 0.5 M sucrose did not lyse the protoplasts while vesicles were slowly pinching off from the vacuoles of protoplasts as indicated by arrowheads. *Scale bars *= 20 μm.

Preliminary trials suggested that the vesicles with adhering P-Chls could be derived from the swelling protoplasts in an osmotic potential-dependent manner. Accordingly, we carried out time-lapse imaging of the vesicle formation from isolated protoplasts subjected to hypo-osmotic shock treatments. We diluted the sucrose concentration of the protoplast culture medium from 0.7 M to various concentrations. When the sucrose concentration was substantial decreased to 0.1 M, the isolated protoplasts expanded rapidly and lysed approximately 15 s after dilution, releasing the CCCs and vesicles with P-Chls (Figure 3A; Additional file 1). The dense CCC structures gradually loosened and the encased C-Chls eventually dissociated from the CCC structures, whereas the vesicles continued to expand beyond the stretching limit of their membrane and eventually bursted causing the P-Chls to adhere to strings of collapsed membrane which were inseparable from the CCC structures (Figure 3A; Additional file 1). On the other hand, when the sucrose concentration was reduced to the ideal 0.2 M, the isolated protoplasts gradually swelled and lysed approximately 25 s after dilution, releasing the CCCs and P-Chl-carrying vesicles (Figure 3B; Addition file 2). Under this condition, the majority of C-Chls remained tightly packed within the dense CCC structures and the vesicles continued to swell for a few seconds yet remained intact after cell lysis (Figure 3B; Additional file 2). Subtle reduction of the sucrose concentration to 0.5 M did not burst the isolated protoplasts, although some vesicles were occasionally pinched off from the intact swelling protoplasts (Figure 3C; Additional file 3). The majority of these vesicles, however, did not carry any chloroplasts (Figure 3C; Additional file 3).

### The vacuolar origin of vesicles from lysed protoplasts

**Figure 4:**
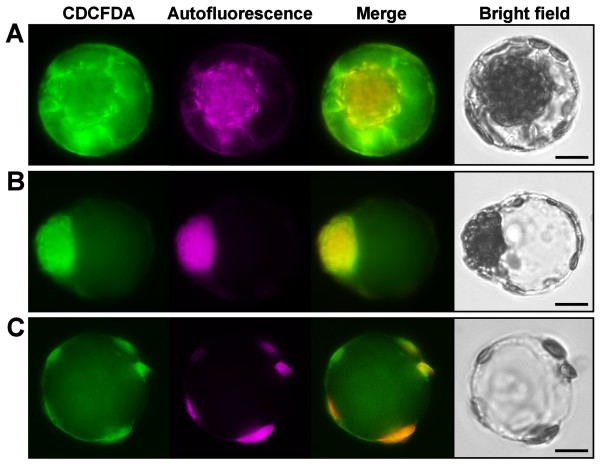
**Vacuolar staining of chlorenchyma protoplasts and osmotically-derived vesicles**. Isolated protoplasts were stained with 5-(and-6)-carboxy-2',7'-dichloro-fluorescein diacetate (CDCFDA) and lysed by osmotic swelling. The isolated protoplasts and induced vesicles were observed under light and epifluorescence microscopy. CDCFDA staining, chlorophyll autofluorescence, merged images of the two channels, and bright field images are shown. (**A**) An isolated protoplast. (**B**) A swelling protoplast before cell lysis. (**C**) A vacuole-derived vesicle. *Scale bars *= 10 μm.

**Figure 5:**
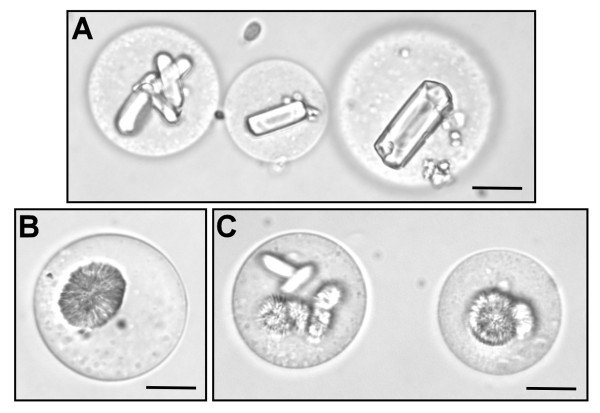
**Formation of calcium oxalate crystals in the vacuole-derived vesicles**. The vesicles were obtained from lysis of protoplasts by osmotic swelling at 0.2 M sucrose and isolated on the floating layer after centrifugation at 100*g*, 4 min. Formation of calcium oxalate crystals was induced by resuspension of the vesicles in cell stabilizing buffer with 0.7 M sucrose. (**A**) A light micrograph of prismatic crystals. (**B**) A light micrograph of raphide crystals. (**C**) A light micrograph of both prismatic and raphide crystals. *Scale bars *= 20 μm.

The time-lapse images clearly showed that the released vesicles from the hypo-osmotically lysed protoplasts originally made up the bulk of cell volume (Figure 3), implying that these vesicles derived from the large central vacuoles. Due to the unusual subcellular organization of *B. sinuspersici *chlorenchyma cells, we sought to confirm the vacuolar origin of the vesicles by staining with 5-(and-6)-carboxy-2',7'-dichloro-fluorescein diacetate (CDCFDA), a pH-sensing vital probe for the vacuole lumen [14]. In a CDCFDA-stained protoplast, prominent fluorescent signals were typically found in the vacuole which occupies the massive cell content separating the CCC and PCC (Figure 4A). Following hypo-osmotic shock treatment of CDCFDA-stained protoplasts, the fluorescent signals persisted in the resulting vesicles indicating their vacuolar origin (Figure 4B and 4C). The occurrence of some patchy CDCFDA signals in other subcellular locations (Figure 4), similar to the observations from previous studies [9,11], might be attributed to the presence of reactive oxygen species [15]. In addition to the CDCFDA staining, the observation of prismatic and raphide crystals in the vesicles provided further evidence of their vacuolar origin (Figure 5). The occurrence of these crystals is a widespread phenomenon in plant vacuoles due to the accumulation of calcium oxalate as crystalline deposits [16].

### Subfractionation of dimorphic chloroplasts from lysed protoplasts

**Figure 6:**
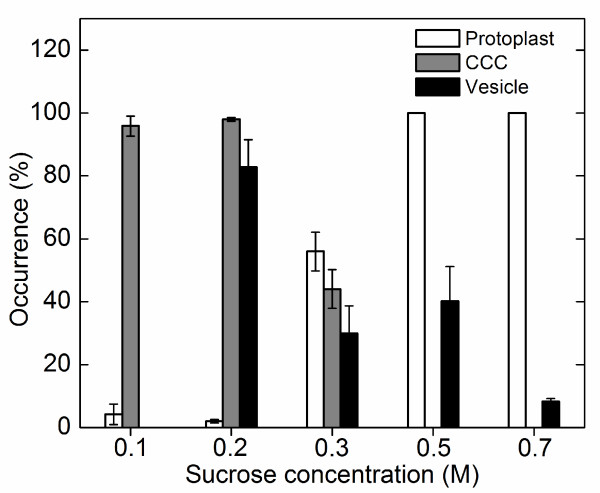
**The release of central cytoplasmic compartments and vesicles from protoplasts under different osmotic conditions**. The sucrose concentration of the cell stabilizing buffer was either undiluted (0.7 M) or diluted to 0.1, 0.2, 0.3 or 0.5 M, and the protoplasts were incubated on ice for 10 min. The number of intact protoplasts, central cytoplasmic compartments (CCC) and vacuole-derived vesicles were counted using a haemocytometer under light microscopy. The occurrence (%) is calculated from the number of protoplasts, CCC or vesicles after osmotic treatment divided by the number of protoplasts used. Each bar represents the mean value from three independent experiments ± 1 standard deviation.

**Figure 7:**
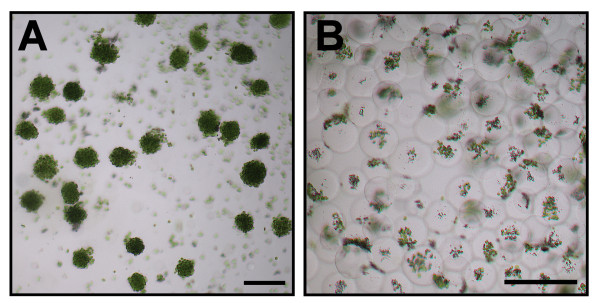
**Bright field images of the two isolated dimorphic chloroplast populations**. (**A**) A homogenous population of central cytoplasmic compartments. (**B**) A homogenous population of vacuole-derived vesicles carrying peripheral chloroplasts on the external surfaces. *Scale bars *= 40 μm.

Given a promising strategy to separate CCCs and intact vacuoles with adhering P-Chls by osmotic treatment of isolated protoplasts, we further optimized the conditions for the best yields of homogenous populations of these two suborganellar structures. Since preliminary trials suggested that the isolated protoplasts under an overcrowded condition responded heterogeneously to the osmotic shock, we routinely adjusted the cell density to 10^5 ^protoplasts mL^-1 ^before the treatment to ensure the highest percentage of protoplast lysis. Pre-conditioning of the isolated protoplasts at 0°C potentially rendered their plasma membrane more brittle due to the reduced fluidity and facilitated the subsequent cell lysis. Rapid dilution of the isolated protoplasts at room temperature in an EDTA-containing buffer without the sucrose osmoticum resulted in stretching and fragmentation of the plasma membrane. This observation is considered a combined effect of protoplast swelling due to the reduced osmotic strength of the buffer and the sudden alternation in membrane fluidity due to the thermal change and the chelation of divalent ions. At the optimum 0.2 M concentration of sucrose, 98% of isolated protoplasts were bursted and ca. 80% of the released vacuoles remained intact (Figure 6). On the other hand, relatively low percentages of protoplasts were lysed at higher sucrose concentrations (i.e. 0.3-0.7 M), whereas the released vacuoles at unfavorably low sucrose concentration (i.e. 0.1 M) were concomitantly bursted (Figure 6). Taking together, we routinely lysed the isolated protoplasts by rapid dilution of the protoplast culture buffer to 0.2 M sucrose which provided the best yields of intact CCCs and intact P-Chl-containing vacuoles. Due to the substantial difference in densities of these two suborganellar structures, subsequent incubation on ice and low-speed centrifugation (100*g*, 4 min) led to their separation into the sedimented and floating fractions, respectively. The recovered pellets and floating layers contained a homogeneous population of CCCs (Figure 7A) and intact vacuoles with adhering P-Chls (Figure 7B), respectively. The CCCs could be easily dispersed mechanically by pipetting up and down following the cold treatment and the C-Chls released from the CCCs were further purified from other impurities on a typical two-step Percoll gradient. By centrifugation (2,500*g*, 10 min) of the P-Chl-containing vacuoles on a similar Percoll gradient, the loosely adhering P-Chls were easily dissociated from the vacuoles and entered the Percoll solutions. In both cases of C-Chls and P-Chls, the lower green band at the 40%/85% interface contained at least 50% of the loaded chloroplasts. The purity and intactness of the aspirated chloroplasts from this interface were confirmed by phase contrast microscopy (data not shown).

### Evaluating cross-contamination of the isolated dimorphic chloroplasts

**Figure 8:**
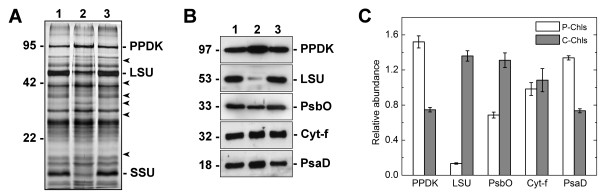
**Silver staining and Western blot analyses of protein extracts from the isolated dimorphic chloroplasts**. Two micrograms of proteins from total chloroplasts (lane 1), peripheral chloroplasts (lane 2) and central chloroplasts (lane 3) were separated on SDS/12% PAGE. Molecular weights are shown on the left in kilodaltons. (**A**) Total proteins were visualized by silver staining. Polypeptides of the highly abundant pyruvate orthophosphate dikinase (PPDK), Rubisco large-subunit (LSU) and small-subunit (SSU) are indicated. Other bands of significantly different intensities between the dimorphic chloroplasts are indicated by arrowheads. (**B**) Resolved proteins were electroblotted onto nitrocellulose membranes and probed with antibodies against PPDK, LSU, Photosystem II manganese-stabilizing protein (PsbO), cytochrome f (Cyt-f), or Photosystem I subunit II (PsaD). (**C**) The immunoblots were analyzed by densitometric quantification. The abundance of each protein in peripheral (P-Chls) and central (C-Chls) chloroplasts was calculated relative to that of total chloroplasts and shown with standard errors, after three (PsbO) or four (others) independent experiments.

The banding patterns of the resolved polypeptides from the isolated dimorphic chloroplasts were visualized and compared by silver staining (Figure 8A). Various differential band intensities were identified by comparing the two protein profiles, with the most striking difference found at the electropheretic mobility regions equivalent to pyruvate orthophosphate dikinase (PPDK; 97 kDa) and the large-subunit (LSU; 53 kDa) and small-subunit (SSU; 14 kDa) of Rubisco (Figure 8A). These protein distribution patterns are in agreement with the recent observations following the subcellular fractionation of P-Chls and C-Chls [10]. In fact, previous immunolocalization experiments also revealed that the labelling of PPDK was mainly associated with the P-Chls, whereas the LSU was predominantly detected in the C-Chls [9]. Western blot analyses further confirmed that the immunoreactive band of PPDK from P-Chls was two-fold more intense than that from C-Chls, whereas LSU was almost exclusively found in C-Chls but barely detectable in P-Chls (Figures 8B, C). The relative distribution of the photosystem I and II proteins in the dimorphic chloroplasts (Figures 8B, C) also correlated with the previous ultrastructural studies which indicated that C-Chls had a higher granal index and generally greater sizes of grana stacks than P-Chls [7]. The Western blot analyses revealed that the C-Chls had slightly more photosystem II manganese-stabilizing proteins (PsbO; Figures 8B, C), which are located predominantly in the stacked regions of thylakoid membrane. The P-Chls, on the other hand, contained more photosystem I subunit II proteins (PsaD; Figures 8B, C), which are commonly found in the unstacked regions. The cytochrome f subunit of the cytochrome b_6_f complex, which is evenly distributed throughout the thylakoid membrane, was equally abundant in both types of chloroplasts (Figures 8B, C). Overall, the electrophoretic analyses indicated minimal cross-contamination of the two isolated populations of dimorphic chloroplasts.

## Discussion

### Potential feasibility of isolating dimorphic chloroplasts from *B. Sinuspersici*

In view of the chlorenchyma cell anatomies of the three currently known terrestrial single-cell C_4 _species, isolation of dimorphic chloroplasts from the two *Bienertia *species is technically more feasible than that from *S. aralocaspica*. Firstly, chlorenchyma cells of the two *Bienertia *species (i.e. *B. sinuspersici *and *B. cycloptera*) enclose one type of chloroplasts within a distinctive spherical structure (CCC) at the centre of the cell and randomly distribute another type of chloroplasts in a thin layer of cytoplasm at the cell periphery (PCC). Separation of the dimorphic chloroplasts from chlorenchyma cells of these species is therefore technically feasible provided that the integrity of CCC is preserved during the process. Secondly, the mature chlorenchyma cells of *Bienertia *species effectively limit gas diffusion by the formation of CCCs in the cell interior, as a means to reduce CO_2 _leakage at the site of C_4 _acid decarboxylation and exposure of Rubisco to O_2_, At the cell periphery, the entire chlorenchyma cells are surrounded by extensive intercellular space to facilitate fixation of atmospheric CO_2 _(Figure 2B) [7]. Therefore, the loosely packed chlorenchyma cells can be readily isolated by gently squeezing the leaves in a mortar and pestle [12]. Technically, this rapid release of chlorenchyma cells devoid of contaminations such as epidermis, water storage and vacuolar tissues facilitates the subsequent isolation of pure and functional dimorphic chloroplasts. In contrast, mature leaves of *S. aralocaspica *partition dimorphic chloroplasts by localization of the first chloroplast type at one pole of the elongated chlorenchyma cells proximal to the atmosphere and the second chloroplast type at the opposite pole of the cells proximal to the vascular tissues [5]. Thus, the operation of the single-cell C_4 _cycle in this species depends primarily on the polarization of the dimorphic chloroplasts and the radial elongation of the cells for effective limitation of CO_2 _and O_2 _diffusion [5,17]. Isolation of the two polarized chloroplast types solely based on the difference in their subcellular locations might be technically challenging, if not impossible. Perhaps, we speculate that any change in cell shape due to cell wall removal, as the first step in routine chloroplast isolation procedures, might distort the polarization of the dimorphic chloroplasts, although the isolation of protoplasts from *S. aralocaspica *chlorenchyma cells has yet been attempted. The leaf anatomy of *S. aralocaspica *also reveals absolute exclusion of intercellular air space toward the inner poles of chlorenchyma cells due to the tight cell-cell adhesion [5,17], implicating the difficulty in chlorenchyma cell isolation. Moreover, our successful plant propagation by vegetative cuttings added another technical advantage of using *B. sinuspersici *leaves for large-scale isolation of dimorphic chloroplasts with synchronized C_4 _development.

### Technical considerations for isolation of central chloroplasts

A reliable protocol for separating the dimorphic chloroplasts is primarily based on the criteria of no cross-contamination. The isolation of C-Chls is relatively effortless due to the confinement of these organelles within the large, dense CCC structures, which can be easily recovered from the cell lysates by low-speed centrifugation (Figure 7A). It is worthy of note, however, that the C-Chl preparation might be potentially contaminated with P-Chls due to incomplete protoplast lysis. Previously, we reported a low-speed centrifugation-based method for floatation of healthy, intact chlorenchyma protoplasts on a sucrose medium and sedimentation of the stressed protoplasts [12]. In the present study, any protoplasts surviving from the osmotically mediated lytic treatment might therefore be co-sedimented with the CCC fraction upon low-speed centrifugation leading to contamination of this fraction with P-Chls. Accordingly, we optimized our procedures for osmotic shock treatment and showed that the isolated protoplasts at a suitable cell density were almost exclusively bursted under the defined chemical, thermal and osmotic conditions (Figure 6), leading to a homogenous preparation (Figure 7A) and satisfactory purity (Figure 8) of C-Chls. In addition to P-Chls, the potential contamination of C-Chl preparation with chloroplasts from immature chlorenchyma cells should also be taken into account. The expression patterns of photosynthetic genes strongly suggested that these chloroplasts from immature chlorenchyma cells, as evident in the young leaf samples, might function in a C_3 _default mode of photosynthetic and photorespiratory pathways [13]. Since no chloroplast clumping was observed at this early stage of leaf development [11], contamination due to immature chlorenchyma cells is not considered a major concern in the preparation of C-Chls using our low-speed centrifugation-based method.

### Technical considerations for isolation of peripheral chloroplasts

On the other hand, purification of P-Chls from the protoplast lysates is deemed technical more challenging. Recently, Offermann et al. [10] reported the first protocol for dimorphic chloroplast isolation from *B. sinuspersici *based on differential speed centrifugation. According to our preliminary study, this centrifugation technique did not provide satisfactory purity of P-Chls due to two major problems. First, residual CCCs remained in the supernatant after the low-speed centrifugation leading to contamination of the P-Chl fraction. Attempts to increase the centrifugal force or duration of centrifugation, on the other hand, resulted in a considerable loss of P-Chls from the supernatant. More remarkably, the supernatant fraction of P-Chls was contaminated with C-Chls due to their dissociation from CCCs, which remains to be an unavoidable problem given the fact that the CCC is not confined by a membrane as revealed at the electron microscopic level (data not shown). In fact, rapid dispersal of C-Chls was commonly observed when the chlorenchyma protoplasts were subjected to unfavorable culture conditions [12] or mechanical disruption such as a gentle press on a microscopic slide [10]. The integrity of CCC structures is primarily maintained by the association of C-Chls in the outer regions of CCCs with the surrounding cytoskeleton networks, of which microtubules have been found to be relatively important [9]. During the dimorphic chloroplast isolation, the instability of the CCCs might be attributed to a combined effect of osmotic swelling of C-Chls in the hypotonic medium and depolymerization of microtubules by low temperature. Despite the contentious issue of cold treatment, chloroplast isolation at higher temperature is not recommended due to the potential risk of protein degradation and denaturation after cell lysis, particularly if chloroplasts are isolated for proteomics or enzymology studies. Similarly, chloroplasts for protein import studies should be isolated at low temperature due to the high susceptibility of the chloroplast protein import receptors to proteolysis [18]. Of relevance to preventing proteolytic degradation, our chloroplast isolation method has an added advantage by confining the majority of proteolytic enzymes in the intact vacuoles.

Due to the considerable contamination of the P-Chls in the cell lysate with dissociated C-Chls, we sought an alternative method for P-Chl purification by isolation of floating vacuoles as P-Chl carriers. The separation of plant vacuoles from isolated protoplasts is a common phenomenon in hypotonic solutions and was documented as early as when protoplasts were isolated for the first time from a plant source [19]. Since then, the protoplast-based techniques for plant vacuole isolation have been well established [20,21]. These isolated vacuoles are commonly surrounded by their tonoplasts with loosely adhering protoplasmic materials [19], including the P-Chls in the present study (Figure 2D). Accordingly, we further optimized the conditions for isolating the vacuoles with adhering P-Chls since the floatation of P-Chls on a sucrose medium might effectively prevent their contamination with CCCs in the pellet or dissociated C-Chls in the supernatant. As expected, our P-Chl preparation using this method barely contained LSU, which was found almost exclusively in the C-Chl fraction as revealed by silver staining and Western blot analysis (Figure 8). The striking 10-fold difference in LSU level between the dimorphic chloroplasts is in agreement with our immunogold quantification analysis at the electron microscopic level showing that the LSU signals were distributed between P-Chls and C-Chls at a ratio of 1:20 (unpublished data). This observation substantially contrasted the previous results obtained using the differential centrifugation method [10]. In their study, Western blot analysis revealed the immunoreactive band intensities of LSU for the P-Chl and C-Chl fractions at a ratio of 1: 3.5 [10], suggesting an impure preparation of P-Chls. Two possible sources of contamination in their P-Chl preparation with LSU might be, as discussed earlier, that the P-Chl fraction was easily contaminated with unpelleted CCCs or dissociated C-Chls using the differential centrifugation method. Also, the C_3_-like chloroplasts from immature chlorenchyma cells were inseparable from the P-Chls because neither type of chloroplasts forms intracellular clumping. On the other hand, since vacuoles were not prominently found in young chlorenchyma cells [11], our preparation of P-Chls using the vacuole-based method was not prone to contamination with C_3_-like chloroplasts, as indicated by the low level of LSU (Figure 8). Moreover, the unequal distribution of proteins associated with the photosystems (PsbO and PsaD) in the two chloroplast fractions presented here also supports earlier ultrastructural results showing that chloroplasts in the PCC have a lower granal index than those in the CCC [7].

### Potential applications

With the development of the current method to isolate two homogenous populations of dimorphic chloroplasts from *B. sinuspersici*, research can be carried out toward a thorough understanding of the single-cell C_4 _mechanism. Enzymology of the C_4 _photosynthetic and photorepiratory pathways can be explicitly characterized. Given the different subsets of enzymes in the two types of chloroplasts, the possibility of differential protein import can be evaluated by *in vitro *import assays and biochemical characterization of the protein import receptors at the chloroplast envelope using the isolated dimorphic chloroplasts. Recently, high-throughput proteomic analyses of the stroma [22] and membrane fractions [23] of the purified mesophyll and bundle sheath cells from maize leaves have provided interesting insights into the different roles of dimorphic chloroplasts in C_4 _photosynthesis as well as other plastid functions. Similar proteomic analysis of the isolated dimorphic chloroplasts from *B. sinuspersici *might allow an informative comparison of their respective roles in single-cell C_4 _photosynthesis and other physiological metabolism. On another perspective, the concomitant isolation of intact vacuoles using the current method might also be useful. For instances, one might further rule out the possibility of crassulacean acid metabolism (CAM) in the single-cell C_4 _species by characterization of the metabolite contents in the vacuoles. Alternatively, the regulation of photosynthetic enzymes in this single-cell C_4 _species might be studied in relation to their turnovers in vacuoles through the autophagic pathway.

## Conclusion

In conclusion, we have established a reliable method for isolating and purifying the dimorphic chloroplasts from a single-cell C_4 _species, *B. sinuspersici*. Following the hypo-osmotic lysis of isolated protoplasts under our defined conditions, one type of chloroplasts can be easily obtained from the discrete subcellular structures in the pellet fraction after low-speed centrifugation, whereas another type of chloroplasts along with vacuoles can be purified by floatation on a sucrose medium using the intact vacuoles as carriers. The purity and homogeneity of the dimorphic chloroplast preparations were evident from the electropheretic analyses of their respective protein markers. This technique can be applied in various experiments to unravel the novel photosynthetic pathways and other physiological aspects in the single-cell C_4 _model.

## Methods

### Isolation of chlorenchyma protoplasts from *B. Sinuspersici *leaves

*B. sinuspersici *plants were propagated asexually by vegetative cuttings essentially as described [12]. Isolation of leaf-derived chlorenchyma protoplasts from *B. sinuspersici *has been optimized previously [12]. Briefly, 20 mature leaves (> 2 cm in length) were freshly harvested from 3- to 4-month-old vegetatively propagated plants and chlorenchyma cells were isolated from the leaves in cell-stabilizing (CS) buffer [0.7 M sucrose, 25 mM HEPES-KOH (pH 6.5), 5 mM KCl and 1 mM CaCl_2_] by gentle pressing using a mortar and pestle. The chlorenchyma cells were collected on a piece of 40-μm nylon mesh filter (Sefar America Inc., USA) with a stack of absorbent paper underneath for removal of the buffer solution. The isolated cells were gently shaken off from the nylon mesh filter into 5 mL of enzyme solution [CS buffer with 1.5% (w/v) cellulase Onozuka R10 (Yakult Honsha Co. Ltd., Tokyo, Japan) and 0.1% (w/v) bovine serum albumin (Sigma-Aldrich, cat. no. A7030)] and incubated at 25°C in the dark without shaking. The progress of cellulase treatment was monitored under light microscopy such that the appearance of round-shaped protoplasts indicates complete cell wall digestion, which was normally achieved within 4 h. The protoplast-washing steps were performed by floating the cells on top of the supernatant after centrifugation at 100*g *for 2 min and gently aspirating them using a micropipette. A homogenous population of chlorenchyma protoplasts was obtained after washing in 5 mL of CS buffer twice. The viability of the isolated protoplasts was assessed by vital staining and the protoplast yield was determined using a Neubauer-Levy haemocytometer (Hausser Scientific, Horsham, PA, USA).

### Separation of dimorphic chloroplasts from Osmotically lysed protoplasts

The isolated protoplasts were washed once in wash buffer [0.7 M sucrose, 25 mM HEPES-KOH (pH 6.5), 5 mM KCl] and resuspended in an appropriate volume of wash buffer to obtain a cell density of 10^5 ^protoplasts mL^-1^. All subsequent steps were carried out at 0~4°C unless otherwise specified. To separate the P-Chls and C-Chls by the hypo-osmotic shock method, 500 μL of protoplast suspension were prechilled on ice for 5 min and then diluted with 1.25 mL of dilution buffer [25 mM HEPES-KOH (pH 6.5), 5 mM KCl, 2 mM EDTA], which had been equilibrated at room temperature, to obtain a final sucrose concentration of 0.2 M. The diluted protoplasts were gently mixed by inverting the tube several times and incubated at room temperature for 2 min. Following the osmotic lysis of protoplasts, the samples were immediately transferred on ice for 10 min with minimal agitation. During this 10-min incubation on ice, the released CCCs were settled to the bottom of the tube while the intact vacuoles carrying P-Chls floated to the top of the medium. The sedimentation of CCCs and the floating of vacuoles with P-Chls were separated by centrifugation at 100*g *for 4 min in a swinging-bucket rotor (A-8-11 for Eppendorf centrifuge 5417R; Eppendorf Canada Ltd., Mississauga, Canada). The floating layer of vacuoles with P-Chls was carefully transferred to a fresh microfuge tube and the aqueous medium was aspirated off with a micropipette without disturbing the pellet containing CCCs. The CCC pellet was washed once in 2 mL of CS buffer by gently tapping with finger, centrifuged at 100*g *for 2 min, and resuspended in 300 μL of dilution buffer. The CCCs were then dissociated by pipetting 10 times up and down using a narrow bore 200-μL micropipette tip. To further purify C-Chls from the other organelles in CCCs, the dissociated CCCs were subjected to Percoll gradient centrifugation as described below. Similar Percoll gradients were used to isolate the loosely associated P-Chls from the vacuole surfaces.

### Percoll gradient purification of chloroplasts

The chloroplast purification procedures were modified according to Smith et al. [24]. Briefly, up to 1 mL of chloroplast suspension was layered on a 40%/85% Percoll step gradient consisting of an upper 500-μL Percoll solution [40% (v/v) Percoll, 50 mM HEPES-KOH (pH 7.3), 330 mM sorbitol, 1 mM MgCl_2_, 1 mM MnCl_2 _and 2 mM EDTA] and a lower 500-μL Percoll solution [85% (v/v) Percoll, 50 mM HEPES-KOH (pH 7.3) and 330 mM sorbitol]. The gradient was centrifuged at 2,500*g*, 4°C for 10 min in a swinging-bucket rotor (A-8-11 for Eppendorf centrifuge 5417R; Eppendorf Canada Ltd., Mississauga, Canada). The intact chloroplasts at the 40%/85% interface of the Percoll gradient were aspirated and diluted with 6 volumes of ice-cold HS buffer [50 mM HEPES-KOH (pH 8.0) and 330 mM sorbitol]. The chloroplasts were pelleted by centrifugation at 750*g*, 4°C for 5 min and resuspended in 50 μL of ice-cold HS buffer.

### Isolation of total chloroplasts from chlorenchyma protoplasts

The procedures for assembling a protoplast-rupturing device were modified according to the method described by Smith et al. [24]. Briefly, the needle-fitting end of a 1-mL syringe barrel and the top part of a 500-μL microtube were cut off to form a hollow tube and a slightly wider adaptor ring, respectively. A piece of 10-μm nylon mesh filter (Spectrum Lab Inc., Rancho Dominguez, CA, USA) was fitted against the cut end of the hollow tube and held in place using the adaptor ring. All subsequent steps were carried out at ~4°C. The isolated protoplasts were resuspended in an appropriate volume of protoplast breakage buffer [20 mM tricine-KOH (pH 8.4), 330 mM sorbitol, 5 mM EDTA, 5 mM EGTA, 10 mM NaHCO_3_] to obtain a cell density of 4 × 10^5 ^protoplasts mL^-1^. Five hundred microliters of protoplast suspension were transferred into the protoplast rupturing device and the plunger was inserted to force the suspension through the mesh. An equal volume of protoplast breakage buffer was then added to the protoplast-rupturing device for completing the breakage of residual protoplasts and CCCs on the mesh. To isolate total chloroplasts from the cell lysate, the pooled filtrates were subjected to Percoll gradient centrifugation as described above.

### SDS-PAGE and western blot analysis

The isolated chloroplasts were resuspended in solubilization buffer [50 mM phosphate buffer (pH 7.2), 1% (w/v) SDS]. The solubilized proteins were quantified using the bicinchoninic acid protein assay kit (Pierce, Rockford, IL, USA) according to the manufacturer's instructions. Two micrograms of proteins were separated on SDS/12% PAGE. The resolved proteins were visualized by silver staining as described previously [25] or electroblotted onto nitrocellulose membranes, which were probed with primary antibodies against PPDK (1:4,000; courtesy of Dr. Chris Chastain), LSU (1:5,000; Agrisera, Vannas, Sweden, cat. no. AS03-037), PsbO (1:5,000; courtesy of Dr. Marilyn Griffith), cytochrome f (1:1,000; Agrisera, Vannas, Sweden, cat. no. AS08-306) or PsaD (1:500; Agrisera, Vannas, Sweden, cat. no. AS09-461), followed by a horseradish peroxidase-conjugated anti-rabbit secondary antibody (1:10,000; Sigma-Aldrich, cat. no. A6154). The signals were visualized by incubating the probed membranes with Amersham ECL-Plus solution (GE Healthcare, cat. no. RPN2132) and exposing the membranes to Amersham Hyperfilm ECL films (GE Healthcare, cat. no. 28-9068-39), which were developed using a CP1000 Agfa photodeveloper (AGFA, Ontario, Canada), digitized and processed using Adobe Photoshop CS (Adobe Systems Inc., Seattle, WA, USA). The intensities of immunoreactive bands were densitometrically quantified using the gel-analyzer function of ImageJ software v.1.46 (National Institutes of Health, USA).

### Cytochemical staining

For vital staining, 200 μL of protoplasts were incubated with 4 μL of 0.2% (w/v) fluorescein diacetate [Sigma-Aldrich, cat. no. F5502; prepared in 100% (v/v) acetone] at room temperature for 15 min and washed twice with 200 μL of CS buffer. For vacuolar staining, 200 μL of protoplasts were centrifuged at 100*g *for 2 min and the floating protoplasts were collected and resuspended in 200 μL of acidic staining buffer [0.7 M sucrose, 50 mM sodium citrate (pH 4), 5 mM KCl and 1 mM CaCl_2_]. The protoplasts were stained with 1 μL of 10 mM CDCFDA [Invitrogen, cat. no. C-369] at room temperature for 15 min and washed twice with 200 μL of acidic staining buffer. The stained protoplasts were observed under epifluorescence microscopy.

### Epifluorescence and light microscopy

Fifty microliters of cytochemically stained protoplasts were examined and imaged in flat-bottomed depression slides under an epifluorescence microscope (Zeiss Axio Imager D1) equipped with a Zeiss AxioCam MRm camera. Leaf samples were fixed, dehydrated and embedded in London Resin White (Electron Microscopy Sciences) acrylic resin as previously described [9]. Semi-thin (1 μm) sections were stained with 1% (w/v) toluidine blue O and observed under light microscopy. Bright field micrographs were acquired using a Zeiss Axiophot microscope (Carl Zeiss Inc., Germany) equipped with a Q-Imaging digital camera (Quorum Technologies Inc., Guelph, Canada). All images were processed and composed using Adobe Photoshop CS (Adobe Systems Inc., Seattle, WA, USA). Representative images were presented after similar results were obtained from at least 3 independent experiments.

## Competing interests

The authors declare that they have no competing interests.

## Authors' contributions

SCL designed and performed the experiments, analyzed the data, and drafted the manuscript. MY performed the electrophoretic analyses. SDXC conceived the study and provided supervision and critical revision of the manuscript. All authors read and approved the final manuscript.

## Supplementary Material

Additional file 1 **An AVI movie showing bursting of protoplasts and their vacuoles at 0.1 M sucrose**. This movie was made from the time-lapse images of protoplasts subjected to osmotic swelling at 0.1 M sucrose. Representative images are shown in Figure 3A. Click here for file

Additional file 2 **An AVI movie showing protoplast lysis and formation of vacuole-derived vesicles at 0.2 M sucrose**. This movie was made from the time-lapse images of protoplasts subjected to osmotic swelling at 0.2 M sucrose. Representative images are shown in Figure 3B. Click here for file

Additional file 3 **An AVI movie showing osmotic swelling of protoplasts and pinching-off of vacuoles at 0.5 M sucrose**. This movie was made from the time-lapse images of protoplasts subjected to osmotic swelling at 0.5 M sucrose. Representative images are shown in Figure 3C. Click here for file
